# Application of discrete choice experiments to enhance stakeholder engagement as a strategy for advancing implementation: a systematic review

**DOI:** 10.1186/s13012-017-0675-8

**Published:** 2017-11-23

**Authors:** Ramzi G. Salloum, Elizabeth A. Shenkman, Jordan J. Louviere, David A. Chambers

**Affiliations:** 1 Department of Health Outcomes and Policy, College of Medicine, University of Florida, 2004 Mowry Road, Gainesville, FL 32610 USA; 2 Institute for Choice, School of Marketing, University of South Australia, Adelaide, SA Australia; 3 Division of Cancer Control and Population Sciences, National Cancer Institute, Rockville, MD USA

**Keywords:** Discrete choice, Conjoint analysis, Preferences, Stakeholder, Engagement

## Abstract

**Background:**

One of the key strategies to successful implementation of effective health-related interventions is targeting improvements in stakeholder engagement. The discrete choice experiment (DCE) is a stated preference technique for eliciting individual preferences over hypothetical alternative scenarios that is increasingly being used in health-related applications. DCEs are a dynamic approach to systematically measure health preferences which can be applied in enhancing stakeholder engagement. However, a knowledge gap exists in characterizing the extent to which DCEs are used in implementation science.

**Methods:**

We conducted a systematic literature search (up to December 2016) of the English literature to identify and describe the use of DCEs in engaging stakeholders as an implementation strategy. We searched the following electronic databases: MEDLINE, Econlit, PsychINFO, and the CINAHL using mesh terms. Studies were categorized according to application type, stakeholder(s), healthcare setting, and implementation outcome.

**Results:**

Seventy-five publications were selected for analysis in this systematic review. Studies were categorized by application type: (1) characterizing demand for therapies and treatment technologies (*n* = 32), (2) comparing implementation strategies (*n* = 22), (3) incentivizing workforce participation (*n* = 11), and (4) prioritizing interventions (*n* = 10). Stakeholders included providers (*n* = 27), patients (*n* = 25), caregivers (*n* = 5), and administrators (*n* = 2). The remaining studies (*n* = 16) engaged multiple stakeholders (i.e., combination of patients, caregivers, providers, and/or administrators). The following implementation outcomes were discussed: acceptability (*n* = 75), appropriateness (*n* = 34), adoption (*n* = 19), feasibility (*n* = 16), and fidelity (*n* = 3).

**Conclusions:**

The number of DCE studies engaging stakeholders as an implementation strategy has been increasing over the past decade. As DCEs are more widely used as a healthcare assessment tool, there is a wide range of applications for them in stakeholder engagement. The DCE approach could serve as a tool for engaging stakeholders in implementation science.

**Electronic supplementary material:**

The online version of this article (10.1186/s13012-017-0675-8) contains supplementary material, which is available to authorized users.

## Background

Implementation science promotes methods to integrate scientific evidence into healthcare practice and policy. Traditionally, it has taken 15–20 years for academic research to translate into evidence-based program and policies, and implementation science is focused on narrowing time for translation of knowledge into practice [1]. One major component of implementation science is stakeholder engagement [2]. Successful implementation of healthcare interventions relies on stakeholder engagement at every stage, ranging from assessing and improving the acceptability of innovations to the sustainability of implemented interventions. In order to optimize the implementation of healthcare interventions, researchers, administrators, and policymakers must weigh the benefits and costs of complex multidimensional arrays of healthcare policies, strategies, and treatments.

As the field of implementation science matures, conceptualizing and measuring implementation outcomes becomes inevitable, particularly as it relates to the context of understanding the demand for evidence-based programs [3, 4]. One strategy for systematically evaluating implementation outcomes involves the assessment of patient health preferences. As a multidisciplinary field, implementation science should leverage health economics tools that assess alternative implementation strategies and communicate the preferences of relevant stakeholders around the characteristics of healthcare programs and interventions.

One dynamic tool for appraising choices in health-related settings is the discrete choice experiment (DCE), which elicits preferences from individual decision makers over alternative scenarios, goods, or services. Each alternative is characterized by several attributes; and the choices subsequently determine how preferences are influenced by each attribute, as well as their relative importance. Health economists increasingly rely on DCEs (also referred to as conjoint analysis) [5] to elicit preferences for healthcare products and programs, which then can be used in outcome measurement for economic evaluation [6]. Despite their utility in improving our understanding of health-related choices, the extent to which DCEs have been applied in implementation research is unknown. In this paper, we explore and document potential applications of DCEs and how these applications can contribute to the field of implementation science by enhancing stakeholder engagement.

There is limited guidance on how to tailor implementation strategies in order to address the contextual needs of change efforts in health-related settings [7]. A recent study identified four methods to improve the selection and tailoring of implementation strategies: (1) concept mapping (i.e., visual mapping using mixed methods); (2) group model building (i.e., causal loop diagrams of complex problems); (3) intervention mapping (i.e., systematic multi-step development of interventions); and (4) DCEs [7]. Although all four methods could be used to match implementation strategies to recognize barriers and facilitators for a particular evidence-based practice or process change being implemented in a given setting, DCEs were identified as having the clear advantages of (1) providing a clear stepwise method for selecting and tailoring strategies to unique settings, while (2) guiding stakeholders to consider attributes of strategies at a granular level, enhancing the precision with which strategies are tailored to context.

Discrete choice experiments are a commonly used technique to address a range of important healthcare questions. DCEs constitute an attribute-based measure of benefit, with the assumptions that first, healthcare interventions, services or policies can be described by their attributes or characteristics and second, the levels of these attributes drive an individual’s valuation of the healthcare good. Within a DCE, respondents are asked to choose between two or more alternatives. The resulting choices reveal an underlying utility function (i.e., an economic measure of preferences over a given set of goods or services). The approach combines econometric analysis with experimental design theory, consumer theory, and random utility theory, which posits that consumers generally choose what they prefer, and where they do not, this can be explained by random factors [6, 8, 9]. Meanwhile, conjoint analysis originated in psychology to address the mathematical representation of the behavior of rankings observed as an outcome of systematic, factorial manipulation of multiple measures. Although there is a distinction between conjoint analysis and DCE, the two terms are used interchangeability by many researchers [5].

Advancing methods to capture stakeholder perspectives is essential for implementation science [10], and consequently, research is needed to document choice experiment methods for assessing the feasibility, acceptability, and validity of stakeholder perspectives. Whereas the use of DCEs in healthcare settings is well documented, there is a knowledge gap in characterizing whether DCE methodology is being applied to improve stakeholder engagement in implementation science. Therefore, the aim of this systematic review was to provide a synthesis of the use of DCEs as a stakeholder engagement tool. Specific objectives were to (1) identify published studies using DCEs in stakeholder engagement; (2) categorize these studies by application subtype, stakeholder group, and healthcare setting; and (3) provide recommendations for future use of DCEs in implementation science.

## Methods

### Identification of eligible publications

To be included, studies must have reported on original research using the DCE methodology and include a discussion of at least one implementation outcome. Studies must also have been available in English and occurred in a health-related setting. Duplicate abstracts were excluded from the review, as were abstracts describing reviews, editorials, commentaries, protocols, conference abstracts, and dissertations.

### Search strategy

A search of MEDLINE, EconLit, PsycINFO, and CINAHL databases was conducted using the following search terms: (“discrete choice” OR “discrete rank” OR “conjoint analysis”) AND (implement*). These four databases were selected as they index journals from the fields of implementation science and include applications of DCEs across a range of health-related contexts or environments including the following: healthcare practice (e.g., clinical, public health, community-based health settings), health policy (e.g., interactions with health decision-makers at local, regional, provincial/state, federal, or international levels), health education (e.g., interactions with health educators in clinical or academic settings), and healthcare administration (e.g., interactions with health system organizations). The keyword search terms were repeated for all four databases. Keyword searches were limited to the English language, covering all published work available up to December 2016 (Additional file 1).

### Coding and data synthesis

Retrieved abstracts were initially assessed against the eligibility criteria by one reviewer (RS) and rejected if the reviewer determined from the title and abstract that the study did not meet inclusion criteria. Full-text copies of the remaining publications were retrieved and further assessed against eligibility criteria to confirm or refute inclusion. Studies meeting the eligibility criteria were then coded by two reviewers. Disagreements were resolved by consensus or by a third reviewer. For all included studies, we recorded the mode of administration (i.e., electronic, paper-based, or via telephone), whether ethics board approval was obtained, the study sponsor, the incentives provided to participants, and the average duration of surveys. Included studies were categorized as follows:

1. Application type: A formative process was used to identify categories of applications used in the studies that met inclusion criteria. All studies were classified according to one of four application types, as follows: (1) characterizing demand for therapies and treatment technologies; (2) comparing implementation strategies; (3) incentivizing workforce participation; and (4) prioritizing interventions. Studies were further coded based on whether implementation science was a primary focus in the research vs. studies that casually discuss one or more implementation outcome.
2. Implementation outcome and stage: All studies were classified based on one more implementation outcomes discussed in the paper. The implementation outcomes were derived from Proctor’s Conceptual Framework for Implementation Outcomes [3, 4] and include acceptability, adoption, appropriateness, feasibility, fidelity, implementation cost, penetration, and sustainability. We also assessed implementation stage—whether early, mid, or late.
3. Stakeholder: All studies were classified according to the stakeholder(s) involved. These included the patient, stakeholder, provider (including physician, nurse, community health worker, and health educator), and administrator (including health system leader, information technology administrator, and policy maker). Sample size (i.e., number of participants in the DCE) was also recorded.
4. Setting: Studies were further classified based on the healthcare setting where the research was conducted, as either primary care (including community-based settings), specialty care, or research that involved the broader health system (including research related to health information technology). Studies were also classified based on the country or countries where the research was conducted. Countries were then categorized as either “high income” or “low and middle income” according to the World Bank income classification [11].

## Results

**Fig. 1:**
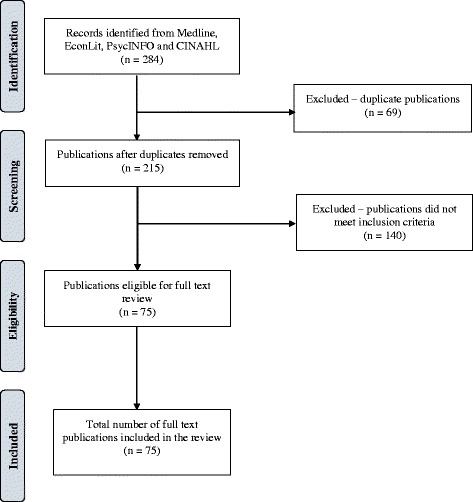
PRISMA flow diagram of study selection

An electronic search yielded a total of 284 titles and abstracts which were judged to be potentially relevant based on title and abstract reading. Of these, 69 records were excluded for being duplicates. Full texts of the remaining 215 articles were reviewed. We finally selected 75 studies that met our inclusion criteria and excluded 140 studies. A flow chart through the different steps of study selection is provided in Fig. 1.

### Excluded studies

A total of 140 studies were excluded. Of these, 47 were not conducted in health-related settings, 38 did not discuss any implementation outcomes, 23 were either commentaries or systematic reviews, 13 were methodological studies without empirical applications, 12 were study protocols, and 7 did not use the DCE methodology. A table with references and reasons for exclusion can be found in Additional file 2.

### Summary of included publications

Of the 75 included studies, 38 were administered as paper-based surveys, 23 were administered electronically, 5 were available in both paper and electronic formats, and 3 were administered via telephone. Administration mode was missing for 6 studies. Overall, 57 studies received institutional review board approval and 38 were exempted.

In terms of sponsorship, 37 studies were supported with government funding, 17 received funding from non-profit organizations, 3 were funded by healthcare delivery systems, and 2 had industry funding. No funding source was listed for the remaining 16 studies. In addition, only 10 studies reported the distribution of financial incentives to participants. The incentives ranged from US$1 or equivalent to US$25 (mean = US$12). Only 7 studies reported the average time it took participants to complete the survey (range 15–30 min).

### Summary of publications over time

**Fig. 2:**
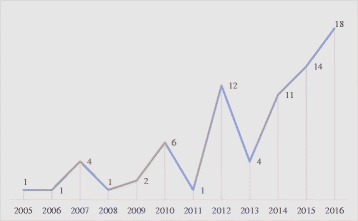
Number of studies, by year: 2005–2016

The earliest DCE study addressing stakeholder engagement in our systematic review was published in 2005. The annual number of publications has steadily increased over the past decade to reach 18 articles in 2016 (Fig. 2).

### Summary of publications by country

**Fig. 3:**
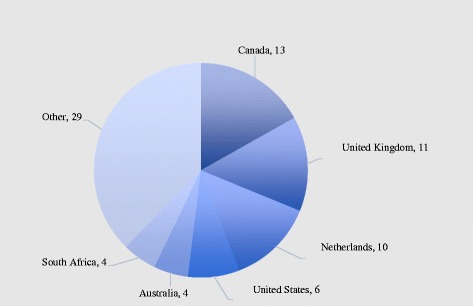
Number of studies, by country

Figure 3 shows the distribution of publications by country. Canada had the largest number of studies that met our inclusion criteria (*n* = 13), followed by the UK (*n* = 11), the Netherlands (*n* = 10), the USA (*n* = 6), Australia (*n* = 4), and South Africa (*n* = 4). Overall, 56 studies were conducted in high-income countries and 19 studies were from low- and middle-income countries (results now shown).

### Summary of publications by healthcare setting

Table 1 shows the number of studies by application type and healthcare setting. The majority of included studies were conducted in the primary care setting (*n* = 46), followed by specialty care (*n* = 22), and across the broader healthcare system (*n* = 7). Primary care studies were distributed as follows, according to application type: characterizing demand for therapies and treatment technologies (*n* = 21); incentivizing workforce participation (*n* = 11); comparing implementations strategies (*n* = 8); and prioritizing interventions (*n* = 4). The most common application in specialty care was comparing implementation strategies (*n* = 12) followed by characterizing demand (*n* = 10). The majority of health system studies focused on prioritizing interventions (*n* = 4), followed by comparing implementation strategies (*n* = 2), and characterizing demand (*n* = 1).

**Table 1:** Summary of studies, by application type and setting

	Setting			
Application type	Health system	Primary care	Specialty care	Total
Characterizing demand for therapies/treatment technologies	1	21	10	32
Comparing implementation strategies	2	8	12	22
Incentivizing workforce participation		11		11
Prioritizing interventions	4	6		10
Total	7	46	22	75

### Summary of publications by stakeholder

Table 2 shows the number of studies by stakeholder type and healthcare setting. A total of 59 studies involved one stakeholder group, distributed as follows: provider (*n* = 27; mean sample size = 408), patient (*n* = 25; mean sample size = 717), caregiver (*n* = 5; mean sample size = 408), and administrator (*n* = 2; mean sample size = 60). The remainder (*n* = 16) involved multiple stakeholders. These were distributed as follows: patient and provider (*n* = 9; mean sample size = 740); provider and administrator (*n* = 3; mean sample size = 532); patient and caregiver (*n* = 2; mean sample size = 492); patient, caregiver, and provider (*n* = 2; mean sample size = 393); and patient, caregiver, provider, and administrator (*n* = 1; samples size = 102). Only 7 of the 46 studies (15%) in primary care involved more than one stakeholder; whereas 9 of 22 studies (41%) in specialty care had multiple stakeholders.

**Table 2:** Summary of studies, by stakeholder and setting

	Setting			Sample size		
Stakeholder	Health system	Primary care	Specialty care	Mean	Range	Total
Patient	3	14	7	717	(35–3372)	24
Caregiver		5		334	(48–820)	5
Provider	2	20	5	408	(45–1720)	27
Administrator	2			60	(41–78)	2
Patient + caregiver		2		492	(112–873)	2
Patient + caregiver + provider			2	393	(224–562)	2
Patient + caregiver + provider + administrator			1	102		1
Patient + provider		3	6	740	(144–3911)	9
Provider + administrator		2	1	532	(66–1379)	3
Total	7	46	22	535	(35–3911)	75

### Summary of publications by implementation outcome

**Fig. 4:**
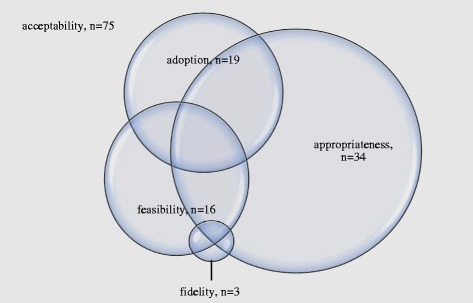
Number of studies, by implementation outcome

Figure 4 shows the documentation of implementation outcomes across the included studies. All included studies were conducted prior to implementation and focused on outcomes associated with early phases of implementation [4]. All 75 publications discussed acceptability. Other outcomes that were discussed include appropriateness (*n* = 34), adoption (*n* = 19), feasibility (*n* = 16), and fidelity (*n* = 3). Outcomes associated with later phases of implementation (i.e., implementation cost, penetration, and sustainability) were not discussed.

### Summary of publications by application type

**Fig. 5:**
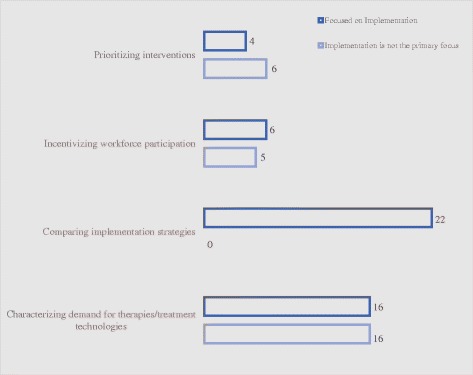
Number of studies, by application type

In terms of application type (Fig. 5), 32 studies were classified as characterizing demand for therapies and treatment technologies [12–42] (16 of 32 [50%] had a primary focus on implementation) [13, 15, 17–19, 21, 22, 25, 26, 29, 33–37, 43]; 22 studies compared implementation strategies [44–65] (22 of 22 [100%] had a primary focus on implementation) [44–65]; 11 studies were concerned with incentivizing workforce participation [66–76] (6 of 11 [55%] had a primary focus on implementation) [66, 68, 71, 74–76]; and 10 studies involved prioritizing health-related interventions [77–86] (4 of 10 [40%] had a primary focus on implementation) [80, 82, 83, 85]. Overall, 48 of the 75 studies (64%) had a primary focus on implementation. The following paragraphs summarize findings by application type:

#### Application 1: characterizing demand for therapies and treatment technologies

Characterizing demand was the most common application among studies included in the current systematic review (*n* = 32) [12–42]. In these studies, decision makers used DCEs in predicting demand for new innovations in healthcare products and services prior to implementation. Because DCEs rely on hypothetical (but realistic) scenarios, they have been used to model the demand for treatment options before they become available to healthcare consumers. Advances in medical technology stipulate that patients and their caregivers choose among alternative scenarios (i.e., traditional therapy vs. new innovation). Forecasting demand for new healthcare technologies has been of great interest to various stakeholders, including public and private payers, healthcare systems, and various health programs and implementing agencies. These studies were overwhelmingly focused on exploring acceptability and appropriateness of health-related product or service, and all 32 of them included the perspectives of either patients or caregivers [12–42].

#### Application 2: comparing implementation strategies

Although the bulk of health-related DCEs examine healthcare preferences and resource allocation, DCEs have also been used in producing decision-making information to guide organizational strategies for implementation of evidence-based practices. Of the 22 studies comparing implementation strategies that were included in the systematic review [44–65], 13 examined the perspective of the provider only [44, 48, 49, 51–54, 57–59, 62, 63], 2 focused only on the patient perspective [56, 60], and 7 examined the perspectives of multiple stakeholders [46, 47, 50, 55, 61, 64, 65]. Furthermore, implementation of patient-centered healthcare provision and the integration of patient priorities into healthcare decision-making require methods for measuring their preferences with respect to health and process outcomes. Therefore, DCEs can be used within the implementation process as tools that elicit stakeholder feedback to ensure the adoption of effective implementation strategies.

#### Application 3: incentivizing workforce participation

Our review found 11 publications that examined the question of incentivizing workforce participation [66–76]. Healthcare providers, including healthcare organizations and the health professionals employed within them, represent key stakeholders in the implementation and delivery of effective interventions. Providers play leading roles in activities that are essential to implementation, including training, supervision, quality assurance, and improvement. All 11 publications in this category took the provider perspective and were conducted in the primary care setting. The range of topics in these studies covered strategies to incentivize community health personnel in low resource settings within low-income countries [66–68, 70, 71, 74, 76] and primary care providers in rural settings within high-income countries [69, 72, 73, 75]. These studies were mainly concerned with investigating the acceptability and appropriateness of their proposed solutions.

#### Application 4: prioritizing delivery of evidence-based interventions

The systematic review found 10 studies that used DCEs to inform the prioritization of health-related interventions [77–86]. Policy makers have long used economic tools, such as cost-effectiveness analysis, to prioritize healthcare service delivery [87]. However, prioritizing healthcare services on the basis of cost-effectiveness alone overlooks other important factors. Among the 10 studies in this category, 4 were conducted at the health system level [79, 82–84] and 6 were in the primary care setting [77, 78, 80, 81, 85, 86]. In terms of stakeholder engagement, 5 studies involved providers [77, 78, 80, 85, 86], 4 involved administrators [77–79, 84], and 3 involved patients [81–83]. This category encompassed a wide range of implementation science topics, including the examination of strategies for approving new medicines in Wales [79], strategies for improving treatment of acute respiratory infections in the USA [86], and priority setting for HIV/AIDS interventions in Thailand [78].

## Discussion

This systematic review identified and synthesized the literature on the use of DCEs to enhance stakeholder engagement as a strategy to improve implementation. Findings suggest that the use of DCE methodology in implementation science has been scarce but growing steadily over the past decade. The current review documented research studies investigating multiple applications of DCEs, namely characterizing demand for therapies and treatment technologies, comparing implementation strategies, incentivizing workforce participation, and prioritizing interventions. The studies were conducted across diverse primary care and specialty care settings and involved several stakeholder groups, including patients, caregivers, providers, and administrators. All studies included in this systematic review were conducted pre-implementation and therefore focused on the investigation of early-stage implementation outcomes (e.g., acceptability and appropriateness).

The systematic review included studies that engaged various stakeholders, including patients, caregivers, providers, and administrators. Successful implementation of evidence-based strategies and programs depend largely on the fit of the interventions with the values and priorities of stakeholders who are shaping and participating in healthcare service delivery and consumption [1]. For example, healthcare recipients and their family members contribute a wide range of perspectives to the evaluation of healthcare services [88], underscoring the importance of systematically assessing their perspectives with respect to evidence-based alternatives. Choosing which evidence-based programs to implement and how to implement them are key decision points for health systems.

Moreover, the preferences of healthcare providers, administrators, and payers within the context of stakeholder engagement inevitably impact the priority attached to healthcare decisions. Effective implementation efforts focusing on individual providers require changes in professional norms and changes in individual providers’ knowledge and beliefs, economic incentives, and other factors [89, 90]. Both financial and non-financial job characteristics can influence the recruitment and retention, as well as the attitudes and perceptions of healthcare professionals toward emerging evidence and innovations. Therefore, understanding the preferences of individual providers can improve the effectiveness of such efforts. However, existing data using revealed preferences are limited in their ability to address provider-level characteristics, and DCEs can be used to better inform this issue [91, 92].

Preference measurement approaches, such as DCEs, are effective instruments for understanding stakeholders’ decision-making. DCEs have been used to engage patients prior to the implementation of cancer screening and tobacco cessation programs [93, 94]. In such studies, researchers were able to gain valuable information about the demand for healthcare services prior to their provision and implementation. Although the choices presented to participants are hypothetical and the responses to them are potentially different from actual behavior, this hypothetical nature has its advantages over actually exposing the participant to the condition, with the researcher having complete control over the experimental design. Combined with advanced statistical techniques, the ability to model hypothetical conditions within the experimental design of DCEs ensures statistical robustness [95]. DCEs also allow the inclusion of attribute levels that do not yet exist, and are ideal for pre-intervention testing. Accordingly, marketing professionals have widely used DCEs in new product development, pricing, market segmentation, and advertising processes [96].

The aforementioned features of DCE studies can be useful in the design of interventions because they can enhance concordance with stakeholder preferences prior to, and during their implementation. The process of integrating research findings into population-level behaviors occurs in context [97]. Context in many healthcare systems includes scarce resources, variability in adoption of existing innovations, and ways of changing behavior that often incur their own costs but are rarely factored into the final estimate of the cost-effectiveness of innovation adoption [98, 99]. DCEs can integrate the assessment of contextual factors, including cost, in the implementation of evidence-based prevention programs.

As the DCE becomes more widely used in healthcare preference assessment, the potential arises for a broad range of applications in implementation science. This systematic review sheds light on the current applications that have been documented in the peer-reviewed literature to date. Implementation science and DCEs are both rapidly emerging concepts in health services research. DCEs are becoming more accepted as an evaluation tool in healthcare while implementation science is now a growing scientific field with funding announcements, annual conferences, training programs, and a growing portfolio of studies globally [100]. Nevertheless, the two areas seldom cross paths. As implementation science advances, there is an opportunity for the field to harness the power of DCEs as a widely accepted tool for engaging stakeholders. The ability of DCEs to present and evaluate attributes and strategies prior to implementation, and their robustness in simultaneously examining these criteria within a decision framework can greatly enhance their value for implementation science.

### Strengths and limitations

To our knowledge, our study is the first to highlight the use of DCEs as a stakeholder engagement strategy to improve implementation. Our study has several strengths, including its explicit and transparent methodology. We conducted a systematic and comprehensive search of the peer-reviewed literature across the relevant databases that resulted in a comprehensive representation of the published research in this area. Further, articles included in this systematic review were categorized into different application types and further classified using the Implementation Outcomes framework to shed light on practical applications for DCEs in implementation science. The identification of these application types and linking them with an implementation science framework provides strong guidance for future studies in this area.

However, our results should be considered in light of several limitations. First, gray literature such as reports, policy documents, and dissertations were not included in the review, nor were protocol papers. Although such reports may be relevant to the topic of interest, gray literature is not peer-reviewed and therefore may not rise to the high standards of quality associated with peer-reviewed publications. Inclusion of gray literature would also have biased the results given that papers related to work known by the authors and their network would have been more likely to have been identified than other works. Second, there are limitations to using volume of research output as a measure of research effort. Due to publication bias, studies with unfavorable results may not be published, leading to under-representation of the actual volume of work carried out in the field. Finally, it is unclear if DCE use effectively influenced implementation strategies and subsequent outcomes, due to the lack of follow-up data in these studies. Whether stakeholder DCEs direct implementation activities to the best approach or outcome remains to be demonstrated in future studies.

## Conclusion

DCEs offer an opportunity to address an underrepresented challenge in implementation science—that of the “demand” side. By bringing key stakeholders to the forefront, we can not only focus on the push of scientific innovations but also understand how best they may be desired, demanded, and valued by patients, families, providers, and administrators. Understanding these dimensions will help us improve how to implement evidence-based interventions and programs in such ways that they will be effectively taken up and the gap in translation of evidence to practice and policy will be shortened.

## Additional files

Additional file 1: List of included studies. (DOCX 40 kb)

Additional file 2: List of excluded studies based on full text evaluation. (DOCX 62 kb)

## Abbreviation

DCE: Discrete choice experiment
