# Supplementary material for: Application of discrete choice experiments to enhance stakeholder engagement as a strategy for advancing implementation: a systematic review
Source: Implement Sci. 2017 Nov 23;12:140. doi: 10.1186/s13012-017-0675-8 (PMC5701380; doi:10.1186/s13012-017-0675-8)
Supplement: Supplementary file 1 — List of included studies. (DOCX 40 kb) [file 13012_2017_675_MOESM1_ESM.docx]

**Additional File 1: Included Studies**

| **IDs** | **References** |
| --- | --- |
| **1** | Determann D, Lambooij MS, Gyrd-Hansen D, et al. Personal health records in the Netherlands: potential user preferences quantified by a discrete choice experiment. *J Am Med Informatics Assoc*. 2016;0(0):ocw158. doi:10.1093/jamia/ocw158. |
| **2** | Uemura H, Matsubara N, Kimura G, et al. Patient preferences for treatment of castration-resistant prostate cancer in Japan: a discrete-choice experiment. *BMC Urol*. 2016;16(1):63. doi:10.1186/s12894-016-0182-2. |
| **3** | Whitty JA, Spinks J, Bucknall T, Tobiano G, Chaboyer W. Patient and nurse preferences for implementation of bedside handover: Do they agree? Findings from a discrete choice experiment. *Heal Expect*. 2016;1(9). doi:10.1111/hex.12513. |
| **4** | Johnson DC, Mueller DE, Deal AM, et al. Integrating Patient Preference into Treatment Decisions for Men with Prostate Cancer at the Point of Care. *J Urol*. 2016;196(6):1640-1644. doi:10.1016/j.juro.2016.06.082. |
| **5** | Gong CL, Hay JW, Meeker D, Doctor JN. Prescriber preferences for behavioural economics interventions to improve treatment of acute respiratory infections: a discrete choice experiment. *BMJ Open*. 2016;6(9):e012739. doi:10.1136/bmjopen-2016-012739. |
| **6** | Ammi M, Peyron C. Heterogeneity in general practitioners’ preferences for quality improvement programs: a choice experiment and policy simulation in France. *Health Econ Rev*. 2016;6(1):44. doi:10.1186/s13561-016-0121-7. |
| **7** | Shiratori S, Agyekum EO, Shibanuma A, et al. Motivation and incentive preferences of community health officers in Ghana: an economic behavioral experiment approach. *Hum Resour Health*. 2016;14(1):53. doi:10.1186/s12960-016-0148-1. |
| **8** | Barrett AN, Advani HV, Chitty LS, et al. Evaluation of preferences of women and healthcare professionals in Singapore for implementation of noninvasive prenatal testing for Down syndrome. *Singapore Med J*. 2016;(June 2016):1-31. doi:10.11622/smedj.2016114. |
| **9** | Morel T, Aymé S, Cassiman D, et al. Quantifying benefit-risk preferences for new medicines in rare disease patients and caregivers. *Orphanet J Rare Dis*. 2016;11(1):70. doi:10.1186/s13023-016-0444-9. |
| **10** | Harrison M, Marra CA, Bansback N. Preferences for “New” Treatments Diminish in the Face of Ambiguity. *Health Economics (United Kingdom)*. http://doi.wiley.com/10.1002/hec.3353. Published 2016. Accessed February 1, 2017. |
| **11** | Herman PM, Ingram M, Cunningham CE, et al. A Comparison of Methods for Capturing Patient Preferences for Delivery of Mental Health Services to Low-Income Hispanics Engaged in Primary Care. *Patient*. 2016;9(4):293-301. doi:10.1007/s40271-015-0155-7. |
| **12** | Chen T-T, Lai M-S, Chung K-P. Participating physician preferences regarding a pay-for-performance incentive design: a discrete choice experiment. *Int J Qual Heal Care*. 2016;28(1):40-46. doi:10.1093/intqhc/mzv098. |
| **13** | Lock J, de Bekker-Grob EW, Urhan G, et al. Facilitating the implementation of pharmacokinetic-guided dosing of prophylaxis in haemophilia care by discrete choice experiment. *Haemophilia*. 2016;22(1):e1-e10. doi:10.1111/hae.12851. |
| **14** | Hill M, Johnson J-A, Langlois S, et al. Preferences for prenatal tests for Down syndrome: an international comparison of the views of pregnant women and health professionals. *Eur J Hum Genet*. 2016;24(7):968-975. doi:10.1038/ejhg.2015.249. |
| **15** | Tang EC, Galea JT, Kinsler JJ, et al. Using conjoint analysis to determine the impact of product and user characteristics on acceptability of rectal microbicides for HIV prevention among Peruvian men who have sex with men. *Sex Transm Infect*. 2016;92(3):200-205. doi:10.1136/sextrans-2015-052028. |
| **16** | Spinks J, Janda M, Soyer HP, Whitty JA. Consumer preferences for teledermoscopy screening to detect melanoma early. *J Telemed Telecare*. 2016;22(1):39-46. doi:10.1177/1357633X15586701. |
| **17** | Becker MPE, Christensen BK, Cunningham CE, et al. Preferences for Early Intervention Mental Health Services: A Discrete-Choice Conjoint Experiment. *Psychiatr Serv*. 2016;67(2):184-191. doi:10.1176/appi.ps.201400306. |
| **18** | Kasteng F, Settumba S, Källander K, et al. Valuing the work of unpaid community health workers and exploring the incentives to volunteering in rural Africa. *Health Policy Plan*. 2016;31(2):205-216. doi:10.1093/heapol/czv042. |
| **19** | Bailey K, Cunningham C, Pemberton J, Rimas H, Morrison KM. Understanding Academic Clinicians’ Decision Making for the Treatment of Childhood Obesity. doi:10.1089/chi.2015.0031. |
| **20** | Grudniewicz A, Bhattacharyya O, Mckibbon KA, Straus SE. Redesigning printed educational materials for primary care physicians: design improvements increase usability. *Implement Sci*. 2012;10:13. doi:10.1186/s13012-015-0339-5. |
| **21** | Powell G, Holmes EAF, Plumpton CO, et al. Pharmacogenetic testing prior to carbamazepine treatment of epilepsy: patients’ and physicians’ preferences for testing and service delivery. *Br J Clin Pharmacol*. 2015;80(5):1149-1159. doi:10.1111/bcp.12715. |
| **22** | Agyei-Baffour P, Boahemaa MY, Addy EA. Contraceptive preferences and use among auto artisanal workers in the informal sector of Kumasi, Ghana: a discrete choice experiment. *Reprod Health*. 2015;12:32. doi:10.1186/s12978-015-0022-y. |
| **23** | Kristian Kjaer N, Halling A, Bjørnskov Pedersen L. General practitioners’ preferences for future continuous professional development: evidence from a Danish discrete choice experiment. *Educ Prim Care*. 2015;26(1):4-10. doi:10.1080/14739879.2015.11494300. |
| **24** | Zickafoose JS, DeCamp LR, Prosser LA, et al. Parents’ Preferences for Enhanced Access in the Pediatric Medical Home. *JAMA Pediatr*. 2015;169(4):358. doi:10.1001/jamapediatrics.2014.3534. |
| **25** | Holte JH, Kjaer T, Abelsen B, Olsen JA. The impact of pecuniary and non-pecuniary incentives for attracting young doctors to rural general practice. *Soc Sci Med*. 2015;128:1-9. doi:10.1016/j.socscimed.2014.12.022. |
| **26** | Hollin IL, Peay HL, Bridges JFP. Caregiver Preferences for Emerging Duchenne Muscular Dystrophy Treatments: A Comparison of Best-Worst Scaling and Conjoint Analysis. *Patient - Patient-Centered Outcomes Res*. 2015;8(1):19-27. doi:10.1007/s40271-014-0104-x. |
| **27** | Paolucci F, Mentzakis E, Defechereux T, Niessen LW. Equity and efficiency preferences of health policy makers in China—a stated preference analysis. *Health Policy Plan*. 2015;30(8):1059-1066. doi:10.1093/heapol/czu123. |
| **28** | Nicaise P, Soto VE, Dubois V, Lorant V. Users’ and Health Professionals’ Values in Relation to a Psychiatric Intervention: The Case of Psychiatric Advance Directives. *Adm Policy Ment Heal Ment Heal Serv Res*. 2015;42(4):384-393. doi:10.1007/s10488-014-0580-2. |
| **29** | Dixon PR, Grant RC, Urbach DR. The Impact of Marketing Language on Patient Preference for Robot-Assisted Surgery. *Surg Innov*. 2015;22(1):15-19. doi:10.1177/1553350614537562. |
| **30** | Honda A, Ryan M, van Niekerk R, McIntyre D. Improving the public health sector in South Africa: eliciting public preferences using a discrete choice experiment. *Health Policy Plan*. 2015;30(5):600-611. doi:10.1093/heapol/czu038. |
| **31** | Fraenkel L, Cunningham M, Peters E. Subjective numeracy and preference to stay with the status quo. *Med Decis Making*. 2015;35(1):6-11. doi:10.1177/0272989X14532531. |
| **32** | Song K, Scott A, Sivey P, Meng Q. Improving Chinese primary care providers’ recruitment and retention: a discrete choice experiment. *Health Policy Plan*. 2015;30(1):68-77. doi:10.1093/heapol/czt098. |
| **33** | Bocoum FY, Koné E, Kouanda S, Yaméogo ME, Bado AR. Which incentive package will retain regionalized health personnel in Burkina Faso: a discrete choice experiment. doi:10.1186/1478-4491-12-S1-S7. |
| **34** | Li J, Scott A, McGrail M, Humphreys J, Witt J. Retaining rural doctors: Doctors’ preferences for rural medical workforce incentives. *Soc Sci Med*. 2014;121:56-64. doi:10.1016/j.socscimed.2014.09.053. |
| **35** | Veldwijk J, Lambooij MS, Bruijning-Verhagen PCJ, Smit HA, Wit GA de. Parental preferences for rotavirus vaccination in young children: A discrete choice experiment. *Vaccine*. 2014;32(47):6277-6283. doi:10.1016/j.vaccine.2014.09.004. |
| **36** | Erdem S, Thompson C. Prioritising health service innovation investments using public preferences: a discrete choice experiment. *Erdem Thompson BMC Heal Serv Res*. 2014;14. doi:10.1186/1472-6963-14-360. |
| **37** | Hl Struik M, Koster F, Schuit AJ, Nugteren R, Veldwijk J, Lambooij MS. The preferences of users of electronic medical records in hospitals: quantifying the relative importance of barriers and facilitators of an innovation. doi:10.1186/1748-5908-9-69. |
| **38** | Deal K, Keshavjee K, Troyan S, Kyba R, Holbrook AM. Physician and patient willingness to pay for electronic cardiovascular disease management. *Int J Med Inform*. 2014;83(7):517-528. doi:10.1016/j.ijmedinf.2014.04.007. |
| **39** | Pechey R, Burge P, Mentzakis E, Suhrcke M, Marteau TM. *Public Acceptability of Population-Level Interventions to Reduce Alcohol Consumption: A Discrete Choice Experiment*. Vol 113.; 2014. doi:10.1016/j.socscimed.2014.05.010. |
| **40** | Baxter J-AB, Roth DE, Al Mahmud A, Ahmed T, Islam M, Zlotkin SH. Tablets Are Preferred and More Acceptable Than Powdered Prenatal Calcium Supplements among Pregnant Women in Dhaka, Bangladesh. *J Nutr*. 2014;144(7):1106-1112. doi:10.3945/jn.113.188524. |
| **41** | Cunningham CE, Barwick M, Short K, et al. Modeling the Mental Health Practice Change Preferences of Educators: A Discrete-Choice Conjoint Experiment. *School Ment Health*. 2014;6(1):1-14. doi:10.1007/s12310-013-9110-8. |
| **42** | Burton CR, Fargher E, Plumpton C, Roberts GW, Owen H, Roberts E. Investigating preferences for support with life after stroke: a discrete choice experiment. *BMC Health Serv Res*. 2014;14(1):63. doi:10.1186/1472-6963-14-63. |
| **43** | Seghieri C, Mengoni A, Nuti S. Applying discrete choice modelling in a priority setting: An investigation of public preferences for primary care models. *Eur J Heal Econ*. 2014;15(7):773-785. doi:10.1007/s10198-013-0542-8. |
| **44** | Deuchert E, Kauer L, Meisen Zannol F. Would you train me with my mental illness? Evidence from a discrete choice experiment. *J Ment Health Policy Econ*. 2013;16(2):67-80. http://www.ncbi.nlm.nih.gov/pubmed/23999204. Accessed February 1, 2017. |
| **45** | Farley K, Thompson C, Hanbury A, Chambers D. Exploring the feasibility of Conjoint Analysis as a tool for prioritizing innovations for implementation. *Implement Sci*. 2013;8:56. doi:10.1186/1748-5908-8-56. |
| **46** | Linley WG, Hughes DA. Decision-makers’ preferences for approving new medicines in wales: A discrete-choice experiment with assessment of external validity. *Pharmacoeconomics*. 2013;31(4):345-355. doi:10.1007/s40273-013-0030-0. |
| **47** | Wheelock A, Eisingerich AB, Ananworanich J, et al. Are Thai MSM Willing to Take PrEP for HIV Prevention? An Analysis of Attitudes, Preferences and Acceptance. *PLoS One*. 2013;8(1):e54288. doi:10.1371/journal.pone.0054288. |
| **48** | Huicho L, Miranda JJ, Diez-Canseco F, et al. Job Preferences of Nurses and Midwives for Taking Up a Rural Job in Peru: A Discrete Choice Experiment. Fort AL, ed. *PLoS One*. 2012;7(12):e50315. doi:10.1371/journal.pone.0050315. |
| **49** | Miranda JJ, Diez-Canseco F, Lema C, et al. Stated Preferences of Doctors for Choosing a Job in Rural Areas of Peru: A Discrete Choice Experiment. Fort AL, ed. *PLoS One*. 2012;7(12):e50567. doi:10.1371/journal.pone.0050567. |
| **50** | Rennie L, Porteous T, Ryan M. Preferences for Managing Symptoms of Differing Severity: A Discrete Choice Experiment. *Value Heal*. 2012;15(8):1069-1076. doi:10.1016/j.jval.2012.06.013. |
| **51** | Hill M, Fisher J, Chitty LS, Morris S. Women’s and health professionals’ preferences for prenatal tests for Down syndrome: a discrete choice experiment to contrast noninvasive prenatal diagnosis with current invasive tests. *Genet Med*. 2012;14(11):905-913. doi:10.1038/gim.2012.68. |
| **52** | Benning TM, Kimman ML, Dirksen CD, Boersma LJ, Dellaert BGC. Combining Individual-Level Discrete Choice Experiment Estimates and Costs to Inform Health Care Management Decisions about Customized Care: The Case of Follow-Up Strategies after Breast Cancer Treatment. *Value Heal*. 2012. doi:10.1016/j.jval.2012.04.007. |
| **53** | Naik-Panvelkar P, Armour C, Rose JM, Saini B. Patient Preferences for Community Pharmacy Asthma Services. *Pharmacoeconomics*. 2012;30(10):961-976. doi:10.2165/11594350-000000000-00000. |
| **54** | Lagarde M, Blaauw D, Cairns J. Cost-effectiveness analysis of human resources policy interventions to address the shortage of nurses in rural South Africa. *Soc Sci Med*. 2012;75(5):801-806. doi:10.1016/j.socscimed.2012.05.005. |
| **55** | Philips H, Mahr D, Remmen R, Weverbergh M, De Graeve D, Van Royen P. Predicting the place of out-of-hours care-A market simulation based on discrete choice analysis. *Health Policy (New York)*. 2012;106(3):284-290. doi:10.1016/j.healthpol.2012.04.010. |
| **56** | Bridges JFP, Searle SC, Selck FW, Martinson NA. Designing family-centered male circumcision services: A conjoint analysis approach. *Patient*. 2012;5(2):101-111. doi:10.2165/11592970-000000000-00000. |
| **57** | Naik-Panvelkar P, Armour C, Rose JM, Saini B. Patients’ value of asthma services in Australian pharmacies: the way ahead for asthma care. *J Asthma*. 2012;49(3):310-316. doi:10.3109/02770903.2012.658130. |
| **58** | Cunningham CE, Henderson J, Niccols A, et al. Preferences for evidence-based practice dissemination in addiction agencies serving women: A discrete-choice conjoint experiment. *Addiction*. 2012;107(8):1512-1524. doi:10.1111/j.1360-0443.2012.03832.x. |
| **59** | Eisingerich AB, Wheelock A, Gomez GB, et al. Attitudes and Acceptance of Oral and Parenteral HIV Preexposure Prophylaxis among Potential User Groups: A Multinational Study. Tachedjian G, ed. *PLoS One*. 2012;7(1):e28238. doi:10.1371/journal.pone.0028238. |
| **60** | Lagarde M, Smith Paintain L, Antwi G, et al. Evaluating Health Workers’ Potential Resistance to New Interventions: A Role for Discrete Choice Experiments. Nosten F, ed. *PLoS One*. 2011;6(8):e23588. doi:10.1371/journal.pone.0023588. |
| **61** | Wen K-Y, Gustafson DH, Hawkins RP, et al. Developing and validating a model to predict the success of an IHCS implementation: the Readiness for Implementation Model. *J Am Med Informatics Assoc*. 2010;17(6):707-713. doi:10.1136/jamia.2010.005546. |
| **62** | Grindrod KA, Marra CA, Colley L, Tsuyuki RT, Lynd LD. Pharmacists’ Preferences for Providing Patient-Centered Services: A Discrete Choice Experiment to Guide Health Policy. *Ann Pharmacother*. 2010;44(10):1554-1564. doi:10.1345/aph.1P228. |
| **63** | Youngkong S, Baltussen R, Tantivess S, Koolman X, Teerawattananon Y. Criteria for priority setting of HIV/AIDS interventions in Thailand: a discrete choice experiment. *BMC Health Serv Res*. 2010;10(1):197-197 1p. doi:10.1186/1472-6963-10-197. |
| **64** | Hinoul P, Goossens A, Roovers JP. Factors determining the adoption of innovative needle suspension techniques with mesh to treat urogenital prolapse: a conjoint analysis study. *Eur J Obstet Gynecol Reprod Biol*. 2010;151(2):212-216. doi:10.1016/j.ejogrb.2010.03.026. |
| **65** | Davison SN, Kromm SK, Currie GR. Patient and health professional preferences for organ allocation and procurement, end-of-life care and organization of care for patients with chronic kidney disease using a discrete choice experiment. *Nephrol Dial Transplant*. 2010;25(7):2334-2341. doi:10.1093/ndt/gfq072. |
| **66** | Bridges JFP, Searle SC, Selck FW, Martinson NA. Engaging Families in the Choice of Social Marketing Strategies for Male Circumcision Services in Johannesburg, South Africa. *Soc Mar Q*. 2010;16(3):60-76. doi:10.1080/15245004.2010.500443. |
| **67** | van Helvoort-Postulart D, van der Weijden T, Dellaert BGC, de Kok M, von Meyenfeldt MF, Dirksen CD. Investigating the complementary value of discrete choice experiments for the evaluation of barriers and facilitators in implementation research: a questionnaire survey. *Implement Sci*. 2009;4:10. doi:10.1186/1748-5908-4-10. |
| **68** | van Helvoort-Postulart D, Dellaert BGC, van der Weijden T, von Meyenfeldt MF, Dirksen CD. Discrete choice experiments for complex health-care decisions: does hierarchical information integration offer a solution? *Health Econ*. 2009;18(8):903-920. doi:10.1002/hec.1411. |
| **69** | Goossens A, Bossuyt PMM, de Haan RJ. Physicians and nurses focus on different aspects of guidelines when deciding whether to adopt them: An application of conjoint analysis. *Med Decis Mak*. 2008;28(1):138-145. doi:10.1177/0272989X07308749. |
| **70** | Fitzpatrick E, Coyle DE, Durieux-Smith A, Graham ID, Angus DE, Gaboury I. Parents’ preferences for services for children with hearing loss: a conjoint analysis study. *Ear Hear*. 2007;28(6):842-849. doi:10.1097/AUD.0b013e318157676d. |
| **71** | Marshall DA, Johnson FR, Phillips KA, Marshall JK, Thabane L, Kulin NA. Measuring patient preferences for colorectal cancer screening using a choice-format survey. *Value Heal*. 2007;10(5):415-430. doi:10.1111/j.1524-4733.2007.00196.x. |
| **72** | Baltussen R, Ten Asbroek AHA, Koolman X, Shrestha N, Bhattarai P, Niessen LW. Priority setting using multiple criteria: Should a lung health programme be implemented in Nepal? *Health Policy Plan*. 2007;22(3):178-185. doi:10.1093/heapol/czm010. |
| **73** | Oudhoff JP, Timmermans DRM, Knol DL, Bijnen AB, Van der Wal G. Prioritising patients on surgical waiting lists: A conjoint analysis study on the priority judgements of patients, surgeons, occupational physicians, and general practitioners. *Soc Sci Med*. 2007;64(9):1863-1875. doi:10.1016/j.socscimed.2007.01.002. |
| **74** | Berchi C, Dupuis JM, Launoy G. The reasons of general practitioners for promoting colorectal cancer mass screening in France. *Eur J Heal Econ*. 2006;7(2):91-98. doi:10.1007/s10198-006-0339-0. |
| **75** | Huis in’t Veld MHA, van Til JA, Ijzerman MJ, Vollenbroek-Hutten MMR. Preferences of general practitioners regarding an application running on a personal digital assistant in acute stroke care. *J Telemed Telecare*. 2005;11 Suppl 1(1_suppl):37-39. doi:10.1258/1357633054461615. |
