# Supplementary material for: Application of discrete choice experiments to enhance stakeholder engagement as a strategy for advancing implementation: a systematic review
Source: Implement Sci. 2017 Nov 23;12:140. doi: 10.1186/s13012-017-0675-8 (PMC5701380; doi:10.1186/s13012-017-0675-8)
Supplement: Supplementary file 2 — List of excluded studies based on full text evaluation. (DOCX 62 kb) [file 13012_2017_675_MOESM2_ESM.docx]

**Additional File 2: Excluded Studies**

| **ID** | **References** | **Reason For Exclusion** |
| --- | --- | --- |
| 1 | Norman R, Kemmler G, Viney R, et al. Order of Presentation of Dimensions Does Not Systematically Bias Utility Weights from a Discrete Choice Experiment. *Value Heal*. 2016;19(8):1033-1038. doi:10.1016/j.jval.2016.07.003. | Not empirical |
| 2 | Rogers AA, Burton MP. Social preferences for the design of biodiversity offsets for shorebirds in Australia. *Conserv Biol*. December 2016. doi:10.1111/cobi.12874. | Not healthcare delivery |
| 3 | Fulop NJ, Ramsay AIG, Vindrola-Padros C, et al. Reorganising specialist cancer surgery for the twenty-first century: a mixed methods evaluation (RESPECT-21). *Implement Sci*. 2016;11(1):155. doi:10.1186/s13012-016-0520-5. | Protocol |
| 4 | Huang Z, Zeng D, Wang J. Factors affecting Chinese broiler farmers’ main preventive practices in response to highly pathogenic avian influenza. *Prev Vet Med*. 2016;134:153-159. doi:10.1016/j.prevetmed.2016.10.013. | Not healthcare delivery |
| 5 | Osman M, Paleti R, Mishra S, Golias MM. Analysis of injury severity of large truck crashes in work zones. *Accid Anal Prev*. 2016;97:261-273. doi:10.1016/j.aap.2016.10.020. | Not healthcare delivery |
| 6 | Tang C, Xu J, Zhang M. The choice and preference for public-private health care among urban residents in China: evidence from a discrete choice experiment. *BMC Health Serv Res*. 2016;16(1):580. doi:10.1186/s12913-016-1829-0. | No implementation strategy |
| 7 | Brown ZS, Kramer RA, Ocan D, Oryema C. Household perceptions and subjective valuations of indoor residual spraying programmes to control malaria in northern Uganda. *Infect Dis poverty*. 2016;5(1):100. doi:10.1186/s40249-016-0190-1. | Not healthcare delivery |
| 8 | Mühlbacher A, Bethge S. First and Foremost Battle the Virus: Eliciting Patient Preferences in Antiviral Therapy for Hepatitis C Using a Discrete Choice Experiment. *Value Heal*. 2016;19(6):776-787. doi:10.1016/j.jval.2016.04.007. | No implementation strategy |
| 9 | Johnson FR, Zhou M. Patient Preferences in Regulatory Benefit-Risk Assessments: A US Perspective. *Value Heal*. 2016;19(6):741-745. doi:10.1016/j.jval.2016.04.008. | Commentary/Systematic Review |
| 10 | Noguera-Artiaga L, Lipan L, Vázquez-Araújo L, Barber X, Pérez-López D, Carbonell-Barrachina ÁA. Opinion of Spanish Consumers on Hydrosustainable Pistachios. *J Food Sci*. 2016;81(10):S2559-S2565. doi:10.1111/1750-3841.13501. | Not healthcare delivery |
| 11 | van Deen WK, Nguyen D, Duran NE, Kane E, van Oijen MGH, Hommes DW. Value redefined for inflammatory bowel disease patients: a choice-based conjoint analysis of patients’ preferences. *Qual Life Res*. 2017;26(2):455-465. doi:10.1007/s11136-016-1398-z. | No implementation strategy |
| 12 | Stipancic J, Zangenehpour S, Miranda-Moreno L, Saunier N, Granié M-A. Investigating the gender differences on bicycle-vehicle conflicts at urban intersections using an ordered logit methodology. *Accid Anal Prev*. 2016;97:19-27. doi:10.1016/j.aap.2016.07.033. | Not healthcare delivery |
|  | Pröbstl-Haider U, Mostegl NM, Kelemen-Finan J, et al. Farmers’ Preferences for Future Agricultural Land Use Under the Consideration of Climate Change. *Environ Manage*. 2016;58(3):446-464. doi:10.1007/s00267-016-0720-4. | Not healthcare delivery |
| 14 | Hunter RF, Brennan SF, Tang J, et al. Effectiveness and cost-effectiveness of a physical activity loyalty scheme for behaviour change maintenance: a cluster randomised controlled trial. *BMC Public Health*. 2016;16(1):618. doi:10.1186/s12889-016-3244-1. | Protocol |
| 15 | Jagger P, Jumbe C. Stoves or sugar? Willingness to adopt improved cookstoves in Malawi. *Energy Policy*. 2016;92:409-419. doi:10.1016/j.enpol.2016.02.034. | Not healthcare delivery |
| 16 | Kindo BP, Wang H, Peña EA. MPBART - Multinomial Probit Bayesian Additive Regression Trees. 2016. https://arxiv.org/pdf/1309.7821.pdf. | Not empirical |
| 17 | Giles EL, Becker F, Ternent L, Sniehotta FF, McColl E, Adams J. Acceptability of Financial Incentives for Health Behaviours: A Discrete Choice Experiment. Mitra N, ed. *PLoS One*. 2016;11(6):e0157403. doi:10.1371/journal.pone.0157403. | No implementation strategy |
| 18 | Adams J, McNaughton RJ, Wigham S, Flynn D, Ternent L, Shucksmith J. Acceptability of Parental Financial Incentives and Quasi-Mandatory Interventions for Preschool Vaccinations: Triangulation of Findings from Three Linked Studies. Bauch CT, ed. *PLoS One*. 2016;11(6):e0156843. doi:10.1371/journal.pone.0156843. | Commentary/Systematic Review |
| 19 | Minh H Van, Chung LH, Giang KB, et al. Potential Impact of Graphic Health Warnings on Cigarette Packages in Reducing Cigarette Demand and Smoking-Related Deaths in Vietnam. *Asian Pac J Cancer Prev*. 2016;17 Suppl:85-90. http://www.ncbi.nlm.nih.gov/pubmed/27087188. | Not healthcare delivery |
| 20 | Klabunde A, Willekens F. Decision-Making in Agent-Based Models of Migration: State of the Art and Challenges. *Eur J Popul*. 2016;32(1):73-97. doi:10.1007/s10680-015-9362-0. | Not healthcare delivery |
| 21 | Turner S, Morris S, Sheringham J, Hudson E, Fulop NJ. Study protocol: DEcisions in health Care to Introduce or Diffuse innovations using Evidence (DECIDE). *Implement Sci*. 2015;11(1):48. doi:10.1186/s13012-016-0412-8. | Protocol |
| 22 | Meyerding SGH. Consumer preferences for food labels on tomatoes in Germany – A comparison of a quasi-experiment and two stated preference approaches. *Appetite*. 2016;103:105-112. doi:10.1016/j.appet.2016.03.025. | Not healthcare delivery |
| 23 | Keane A, Gurd H, Kaelo D, et al. Gender Differentiated Preferences for a Community-Based Conservation Initiative. Baldwin RF, ed. *PLoS One*. 2016;11(3):e0152432. doi:10.1371/journal.pone.0152432. | Not healthcare delivery |
| 24 | Annunziata A, Pomarici E, Vecchio R, Mariani A. Nutritional information and health warnings on wine labels: Exploring consumer interest and preferences. *Appetite*. 2016;106:58-69. doi:10.1016/j.appet.2016.02.152. | Not healthcare delivery |
| 25 | Mühlbacher AC, Bridges JFP, Bethge S, et al. Preferences for antiviral therapy of chronic hepatitis C: a discrete choice experiment. *Eur J Heal Econ*. 2017;18(2):155-165. doi:10.1007/s10198-016-0763-8. | No implementation strategy |
| 26 | Taylor WJ. Pros and cons of conjoint analysis of discrete choice experiments to define classification and response criteria in rheumatology. *Curr Opin Rheumatol*. 2016;28(2):117-121. doi:10.1097/BOR.0000000000000259. | Commentary/Systematic Review |
| 27 | Mühlbacher AC, Kaczynski A, Zweifel P, Johnson FR. Experimental measurement of preferences in health and healthcare using best-worst scaling: an overview. *Health Econ Rev*. 2016;6(1):2. doi:10.1186/s13561-015-0079-x. | Commentary/Systematic Review |
| 28 | Kim Y, Radoias V. Education, individual time preferences, and asymptomatic disease detection. *Soc Sci Med*. 2016;150:15-22. doi:10.1016/j.socscimed.2015.11.051. | Not healthcare delivery |
| 29 | Arimura TH, Darnall N, Ganguli R, Katayama H. The effect of ISO 14001 on environmental performance: Resolving equivocal findings. *J Environ Manage*. 2016;166:556-566. doi:10.1016/j.jenvman.2015.10.032. | Not healthcare delivery |
| 30 | Norman R, Viney R, Aaronson NK, et al. Using a discrete choice experiment to value the QLU-C10D: feasibility and sensitivity to presentation format. *Qual Life Res*. 2016;25(3):637-649. doi:10.1007/s11136-015-1115-3. | Not healthcare delivery |
| 31 | Coast J, Huynh E, Kinghorn P, Flynn T. Complex Valuation: Applying Ideas from the Complex Intervention Framework to Valuation of a New Measure for End-of-Life Care. *Pharmacoeconomics*. 2016;34(5):499-508. doi:10.1007/s40273-015-0365-9. | No implementation strategy |
| 32 | Schaarschmidt M-L, Kromer C, Herr R, et al. Patient Preferences for Biologicals in Psoriasis: Top Priority of Safety for Cardiovascular Patients. Khodarahmi R, ed. *PLoS One*. 2015;10(12):e0144335. doi:10.1371/journal.pone.0144335. | No implementation strategy |
| 33 | Wang Y, Tong L, Jiang M, Zheng J. Non-rigid structure estimation in trajectory space from monocular vision. *Sensors (Basel)*. 2015;15(10):25730-25745. doi:10.3390/s151025730. | Not healthcare delivery |
| 34 | Schreiner JA, Latacz-Lohmann U. Farmers’ valuation of incentives to produce genetically modified organism-free milk: Insights from a discrete choice experiment in Germany. *J Dairy Sci*. 2015;98(11):7498-7509. doi:10.3168/jds.2015-9515. | Not healthcare delivery |
| 35 | Powell BJ, Beidas RS, Lewis CC, et al. Methods to Improve the Selection and Tailoring of Implementation Strategies. *J Behav Health Serv Res*. August 2015:1-18. doi:10.1007/s11414-015-9475-6. | Commentary/Systematic Review |
| 36 | Reeb PD, Bramardi SJ, Steibel JP, Jaffrézic F, Jeanmougin M, Servant N. Assessing Dissimilarity Measures for Sample-Based Hierarchical Clustering of RNA Sequencing Data Using Plasmode Datasets. Jordan IK, ed. *PLoS One*. 2015;10(7):e0132310. doi:10.1371/journal.pone.0132310. | Not healthcare delivery |
| 37 | Andreopoulos D, Damigos D, Comiti F, Fischer C. Handling preference heterogeneity for river services’ adaptation to climate change. *J Environ Manage*. 2015;160:201-211. doi:10.1016/j.jenvman.2015.06.015. | Not healthcare delivery |
| 38 | Kromer C, Schaarschmidt M-L, Schmieder A, Herr R, Goerdt S, Peitsch WK. Patient Preferences for Treatment of Psoriasis with Biologicals: A Discrete Choice Experiment. *PLoS One*. 2015;10(6):e0129120. doi:10.1371/journal.pone.0129120. | No implementation strategy |
| 39 | Pacou M, Basso F, Gore C, et al. Patient and physician preferences for the treatment of chronic hepatitis C virus infections. *Eur J Gastroenterol Hepatol*. 2015;27(9):1. doi:10.1097/MEG.0000000000000410. | No implementation strategy |
| 40 | Lorenz N. The interaction of direct and indirect risk selection. *J Health Econ*. 2015;42:81-89. doi:10.1016/j.jhealeco.2014.12.003. | Not empirical |
| 41 | Ryan M, Yi D, Avenell A, et al. Gaining pounds by losing pounds: preferences for lifestyle interventions to reduce obesity. *Heal Econ Policy Law*. 2015;10(2):161-182. doi:10.1017/S1744133114000413. | No implementation strategy |
| 42 | Mühlbacher AC, Junker U, Juhnke C, et al. Chronic pain patients’ treatment preferences: a discrete-choice experiment. *Eur J Heal Econ*. 2015;16(6):613-628. doi:10.1007/s10198-014-0614-4. | Not healthcare delivery |
| 43 | Robert M, Hu W, Nielsen MK, Stowe CJ. Attitudes towards implementation of surveillance-based parasite control on Kentucky Thoroughbred farms - Current strategies, awareness and willingness-to-pay. *Equine Vet J*. 2015;47(6):694-700. doi:10.1111/evj.12344. | No implementation strategy |
| 44 | Spinks J, Chaboyer W, Bucknall T, Tobiano G, Whitty JA. Patient and nurse preferences for nurse handover-using preferences to inform policy: a discrete choice experiment protocol. *BMJ Open*. 2015;5(11):e008941. doi:10.1136/bmjopen-2015-008941. | Not empirical |
| 45 | De Brún A, Flynn D, Joyce K, et al. Understanding clinicians’ decisions to offer intravenous thrombolytic treatment to patients with acute ischaemic stroke: a protocol for a discrete choice experiment. *BMJ Open*. 2014;4(7):e005612. doi:10.1136/bmjopen-2014-005612. | Protocol |
| 46 | Clark MD, Determann D, Petrou S, Moro D, de Bekker-Grob EW. Discrete Choice Experiments in Health Economics: A Review of the Literature. *Pharmacoeconomics*. 2014;32(9):883-902. doi:10.1007/s40273-014-0170-x. | Commentary/Systematic Review |
| 47 | Oppe M, Devlin NJ, van Hout B, Krabbe PFM, de Charro F. A Program of Methodological Research to Arrive at the New International EQ-5D-5L Valuation Protocol. *Value Heal*. 2014;17(4):445-453. doi:10.1016/j.jval.2014.04.002. | Not empirical |
| 48 | Ratcliffe J, Lancsar E, Luszcz M, et al. A health economic model for the development and evaluation of innovations in aged care: an application to consumer-directed care-study protocol. *BMJ Open*. 2014;4(6):e005788. doi:10.1136/bmjopen-2014-005788. | Protocol |
| 49 | Abiiro GA, Leppert G, Mbera GB, Robyn PJ, De Allegri M. Developing attributes and attribute-levels for a discrete choice experiment on micro health insurance in rural Malawi. *BMC Health Serv Res*. 2014;14(1):235. doi:10.1186/1472-6963-14-235. | Protocol |
| 50 | Wortley S, Wong G, Kieu A, Howard K. Assessing Stated Preferences for Colorectal Cancer Screening: A Critical Systematic Review of Discrete Choice Experiments. *Patient - Patient-Centered Outcomes Res*. 2014;7(3):271-282. doi:10.1007/s40271-014-0054-3. | Commentary/Systematic Review |
| 51 | Ojakaa D, Olango S, Jarvis J. Factors affecting motivation and retention of primary health care workers in three disparate regions in Kenya. *Hum Resour Health*. 2014;12(1):33. doi:10.1186/1478-4491-12-33. | Not DCE |
| 52 | Michaels-Igbokwe C, Lagarde M, Cairns J, Terris-Prestholt F. Using decision mapping to inform the development of a stated choice survey to elicit youth preferences for sexual and reproductive health and HIV services in rural Malawi. *Soc Sci Med*. 2014;105:93-102. doi:10.1016/j.socscimed.2014.01.016. | Not healthcare delivery |
| 53 | Soliño M, Farizo BA, Louviere J, Jones R. Personal Traits Underlying Environmental Preferences: A Discrete Choice Experiment. Houser D, ed. *PLoS One*. 2014;9(2):e89603. doi:10.1371/journal.pone.0089603. | Protocol |
| 54 | Hill M, Suri R, Nash E, Morris S, Chitty L. Preferences for Prenatal Tests for Cystic Fibrosis: A Discrete Choice Experiment to Compare the Views of Adult Patients, Carriers of Cystic Fibrosis and Health Professionals. *J Clin Med*. 2014;3(1):176-190. doi:10.3390/jcm3010176. | No implementation strategy |
| 55 | Hoefman RJ, van Exel J, Rose JM, van de Wetering EJ, Brouwer WBF. A Discrete Choice Experiment to Obtain a Tariff for Valuing Informal Care Situations Measured with the CarerQol Instrument. *Med Decis Mak*. 2014;34(1):84-96. doi:10.1177/0272989X13492013. | Not empirical |
| 56 | Viney R, Norman R, Brazier J, et al. An Australian Discrete Choice Experiment to Value EQ-5D health status. *Health Econ*. 2014;23(6):729-742. doi:10.1002/hec.2953. | Not empirical |
| 57 | Hoefman RJ, van Exel J, Rose JM, van de Wetering EJ, Brouwer WBF. A Discrete Choice Experiment to Obtain a Tariff for Valuing Informal Care Situations Measured with the CarerQol Instrument. *Med Decis Mak*. 2014;34(1):84-96. doi:10.1177/0272989X13492013. | Not empirical |
| 58 | Mühlbacher AC, Stoll M, Mahlich J, Nübling M. Evaluating the concordance of physician judgments and patient preferences on AIDS/HIV therapy - a Discrete Choice Experiment. *Health Econ Rev*. 2013;3(1):30. doi:10.1186/2191-1991-3-30. | No implementation strategy |
| 59 | Masozera M, Erickson JD, Clifford D, Coppolillo P, Sadiki HG, Mazet JK. Integrating the Management of Ruaha Landscape of Tanzania with Local Needs and Preferences. *Environ Manage*. 2013;52(6):1533-1546. doi:10.1007/s00267-013-0175-9. | Not healthcare delivery |
| 60 | Giraud G, Amblard C, Thiel E, et al. A cross-cultural segmentation of western Balkan consumers: focus on preferences toward traditional fresh cow cheese. *J Sci Food Agric*. 2013;93(14):3464-3472. doi:10.1002/jsfa.6350. | Not healthcare delivery |
| 61 | Slankamenac K, Graf R, Barkun J, Puhan MA, Clavien P-A. The Comprehensive Complication Index. *Ann Surg*. 2013;258(1):1-7. doi:10.1097/SLA.0b013e318296c732. | Not empirical |
| 62 | Kurzyski PL, Wójcik A. Quantum walk as a generalized measuring device. 2012. https://arxiv.org/pdf/1208.1800.pdf. | Not empirical |
| 63 | Norman R, Cronin P, Viney R. A Pilot Discrete Choice Experiment to Explore Preferences for EQ-5D-5L Health States. *Appl Health Econ Health Policy*. 2013;11(3):287-298. doi:10.1007/s40258-013-0035-z. | Not empirical |
| 64 | Laba T-L, Brien J, Fransen M, Jan S. Patient preferences for adherence to treatment for osteoarthritis: the MEdication Decisions in Osteoarthritis Study (MEDOS). *BMC Musculoskelet Disord*. 2013;14(1):160. doi:10.1186/1471-2474-14-160. | No implementation strategy |
| 65 | McNamara A, Chen G, George S, Walker R, Ratcliffe J. What factors influence older people in the decision to relinquish their driver’s licence? A discrete choice experiment. *Accid Anal Prev*. 2013;55:178-184. doi:10.1016/j.aap.2013.02.034. | Not healthcare delivery |
| 66 | Parkinson B, Goodall S, Norman R. Measuring the Loss of Consumer Choice in Mandatory Health Programmes Using Discrete Choice Experiments. *Appl Health Econ Health Policy*. 2013;11(2):139-150. doi:10.1007/s40258-013-0017-1. | No implementation strategy |
| 67 | Johnson FR, Lancsar E, Marshall D, et al. Constructing Experimental Designs for Discrete-Choice Experiments: Report of the ISPOR Conjoint Analysis Experimental Design Good Research Practices Task Force. doi:10.1016/j.jval.2012.08.2223. | Commentary/Systematic Review |
| 68 | Naik-Panvelkar P, Armour C, Saini B. Discrete choice experiments in pharmacy: a review of the literature. *Int J Pharm Pract*. 2013;21(1):3-19. doi:10.1111/ijpp.12002. | Commentary/Systematic Review |
| 69 | Marsh K, Dolan P, Kempster J, Lugon M. Prioritizing investments in public health: a multi-criteria decision analysis. *J Public Health (Bangkok)*. 2013;35(3):460-466. doi:10.1093/pubmed/fds099. | No implementation strategy |
| 70 | Mehta G, Lou Y. Modeling school bus seat belt usage: Nested and mixed logit approaches. *Accid Anal Prev*. 2013;51:56-67. doi:10.1016/j.aap.2012.10.008. | Not healthcare delivery |
| 71 | Krucien N, Gafni A, Fleury B, Pelletier-Fleury N. Patients’ with obstructive sleep apnoea syndrome (OSAS) preferences and demand for treatment: a discrete choice experiment. *Thorax*. 2013;68(5):487-488. doi:10.1136/thoraxjnl-2012-202240. | No implementation strategy |
| 72 | Hoffenson S, Frischknecht BD, Papalambros PY. A market systems analysis of the U.S. Sport Utility Vehicle market considering frontal crash safety technology and policy. *Accid Anal Prev*. 2013;50:943-954. doi:10.1016/j.aap.2012.07.021. | Not healthcare delivery |
| 73 | Norman R, Hall J, Street D, Viney R. EFFICIENCY AND EQUITY: A STATED PREFERENCE APPROACH. *Health Econ*. 2013;22(5):568-581. doi:10.1002/hec.2827. | No implementation strategy |
| 74 | Saywell N, Vandal AC, Brown P, et al. Telerehabilitation to improve outcomes for people with stroke: study protocol for a randomised controlled trial. *Trials*. 2012;13(1):233. doi:10.1186/1745-6215-13-233. | Protocol |
| 75 | Diorio C, Tomlinson D, Boydell KM, et al. Attitudes toward Infection Prophylaxis in Pediatric Oncology: A Qualitative Approach. Moormann AM, ed. *PLoS One*. 2012;7(10):e47815. doi:10.1371/journal.pone.0047815. | Not DCE |
| 76 | Kauf TL, Mohamed AF, Hauber AB, Fetzer D, Ahmad A. Patientsʼ Willingness to Accept the Risks and Benefits of New Treatments for Chronic Hepatitis C Virus Infection. *Patient Patient-Centered Outcomes Res*. 2012;5(4):265-278. doi:10.2165/11633580-000000000-00000. | No implementation strategy |
| 77 | Poulos C, Yang J-C, Patil SR, et al. Consumer preferences for household water treatment products in Andhra Pradesh, India. *Soc Sci Med*. 2012;75(4):738-746. doi:10.1016/j.socscimed.2012.02.059. | Not healthcare delivery |
| 78 | Bowen A, Hesketh A, Patchick E, et al. Clinical effectiveness, cost-effectiveness and service users’ perceptions of early, well-resourced communication therapy following a stroke: a randomised controlled trial (the ACT NoW Study). *Health Technol Assess (Rockv)*. 2012;16(26):1-160. doi:10.3310/hta16260. | No implementation strategy |
| 79 | Fujitani ML, Fenichel EP, Torre J, Gerber LR. Implementation of a marine reserve has a rapid but short-lived effect on recreational angler use. *Ecol Appl*. 2012;22(2):597-605. http://www.ncbi.nlm.nih.gov/pubmed/22611857. | Not healthcare delivery |
| 80 | Veitch C, Lincoln M, Bundy A, et al. Integrating evidence into policy and sustainable disability services delivery in western New South Wales, Australia: the “wobbly hub and double spokes” project. *BMC Health Serv Res*. 2012;12(1):70. doi:10.1186/1472-6963-12-70. | Protocol |
| 81 | Lim MK, Bae EY, Choi S-E, Lee EK, Lee T-J. Eliciting Public Preference for Health-Care Resource Allocation in South Korea. *Value Heal*. 2012;15(1):S91-S94. doi:10.1016/j.jval.2011.11.014. | Not healthcare delivery |
| 82 | Yang X, Regan K, Huang Y, et al. Single Sample Expression-Anchored Mechanisms Predict Survival in Head and Neck Cancer. Dunbrack RL, ed. *PLoS Comput Biol*. 2012;8(1):e1002350. doi:10.1371/journal.pcbi.1002350. | No implementation strategy |
| 83 | Tuunainen P, Valaja J, Valkonen E, Hepola H. Using Conjoint Analysis to Weight Broiler Welfare Variables Based on Slaughterhouse Data. *J Appl Anim Welf Sci*. 2012;15(1):70-79. doi:10.1080/10888705.2011.600666. | Not healthcare delivery |
| 84 | Cimaroli K, Páez A, Bruce Newbold K, Heddle NM. Individual and contextual determinants of blood donation frequency with a focus on clinic accessibility: A case study of Toronto, Canada. *Health Place*. 2012;18(2):424-433. doi:10.1016/j.healthplace.2011.12.005. | Not DCE |
| 85 | Gifford E V. Commentary on Cunningham *et al* . (2012): Benefit to clients - outcome monitoring and knowledge translation. *Addiction*. 2012;107(8):1525-1526. doi:10.1111/j.1360-0443.2012.03964.x. | Commentary/Systematic Review |
| 86 | Gabay G, Moskowitz HR. The algebra of health concerns: implications of consumer perception of health loss, illness and the breakdown of the health system on anxiety. *Int J Consum Stud*. 2012;36(6):635-646. doi:10.1111/j.1470-6431.2011.01038.x. | Commentary/Systematic Review |
| 87 | Cunningham CE, Bruce BS, Snowdon AW, et al. Modeling improvements in booster seat use: A discrete choice conjoint experiment. *Accid Anal Prev*. 2011;43(6):1999-2009. doi:10.1016/j.aap.2011.05.018. | Not healthcare delivery |
| 88 | Twijnstra ARH, Stiggelbout AM, de Kroon CD, Jansen FW. Laparoscopic Hysterectomy: Eliciting Preference of Performers and Colleagues Via Conjoint Analysis. *J Minim Invasive Gynecol*. 2011;18(5):582-588. doi:10.1016/j.jmig.2011.05.009 | No implementation strategy |
| 89 | Poulos C, Yang J-C, Levin C, Van Minh H, Giang KB, Nguyen D. Mothers’ preferences and willingness to pay for HPV vaccines in Vinh Long Province, Vietnam. *Soc Sci Med*. 2011;73(2):226-234. doi:10.1016/j.socscimed.2011.05.029. | No implementation strategy |
| 90 | de Silva-Sanigorski AM, Waters E, Calache H, et al. Splash!: a prospective birth cohort study of the impact of environmental, social and family-level influences on child oral health and obesity related risk factors and outcomes. *BMC Public Health*. 2011;11(1):505. doi:10.1186/1471-2458-11-505. | Protocol |
| 91 | Balana BB, Yatich T, Mäkelä M. A conjoint analysis of landholder preferences for reward-based land-management contracts in Kapingazi watershed, Eastern Mount Kenya. *J Environ Manage*. 2011;92(10):2634-2646. doi:10.1016/j.jenvman.2011.06.001. | Not healthcare delivery |
| 92 | Jaeger SR, Harker R, Triggs CM, et al. Determining Consumer Purchase Intentions: The Importance of Dry Matter, Size, and Price of Kiwifruit. *J Food Sci*. 2011;76(3):S177-S184. doi:10.1111/j.1750-3841.2011.02084.x. | Not healthcare delivery |
| 93 | Santos AC, Roberts JA, Barreto ML, Cairncross S. Demand for sanitation in Salvador, Brazil: A hybrid choice approach. *Soc Sci Med*. 2011;72(8):1325-1332. doi:10.1016/j.socscimed.2011.02.018. | Not healthcare delivery |
| 94 | Bridges JFP, Selck FW, Gray GE, McIntyre JA, Martinson NA. Condom avoidance and determinants of demand for male circumcision in Johannesburg, South Africa. *Health Policy Plan*. 2011;26(4):298-306. doi:10.1093/heapol/czq064. | No implementation strategy |
| 95 | Garro LC. Enacting Ethos, Enacting Health: Realizing Health in the Everyday Life of a California Family of Mexican Descent. *Ethos*. 2011;39(3):300-330. doi:10.1111/j.1548-1352.2011.01195.x. | Not DCE |
| 96 | Jaskiewicz W, Tulenko K, Rockers P, Wurts L, Mgomella G. Retaining hospital workers: a rapid methodology to determine incentive packages. *World Hosp Health Serv*. 2010;46(3):8-11. http://www.ncbi.nlm.nih.gov/pubmed/21155422. | Protocol |
| 97 | Utens CM, Goossens LM, Smeenk FW, et al. Effectiveness and cost-effectiveness of early assisted discharge for Chronic Obstructive Pulmonary Disease exacerbations: the design of a randomised controlled trial. *BMC Public Health*. 2010;10(1):618. doi:10.1186/1471-2458-10-618. | Protocol |
| 98 | Marshall D, McGregor SE, Currie G. Measuring Preferences for Colorectal Cancer Screening. *Patient Patient-Centered Outcomes Res*. 2010;3(2):79-89. doi:10.2165/11532250-000000000-00000. | Commentary/Systematic Review |
| 99 | Flynn TN. Using Conjoint Analysis and Choice Experiments to Estimate QALY Values. *Pharmacoeconomics*. 2010;28(9):711-722. doi:10.2165/11535660-000000000-00000. | Commentary/Systematic Review |
| 100 | Benaïm C, Perennou D-A, Pelissier J-Y, Daures J-P. Using an analytical hierarchy process (AHP) for weighting items of a measurement scale: A pilot study. *Rev Epidemiol Sante Publique*. 2010;58(1):59-63. doi:10.1016/j.respe.2009.09.004. | Not empirical |
| 101 | Fraga AMA, Fraga GP, Noordenbos J, et al. Beach and Campfire Burns: A Site of Pleasure and Tragedy. *J Burn Care Res*. 2010;31(1):184-189. doi:10.1097/BCR.0b013e3181c7ed46. | Not DCE |
| 102 | Newman PA, Roungprakhon S, Tepjan S, Yim S. Preventive HIV vaccine acceptability and behavioral risk compensation among high-risk men who have sex with men and transgenders in Thailand. *Vaccine*. 2010;28(4):958-964. doi:10.1016/j.vaccine.2009.10.142. | No implementation strategy |
| 103 | Yin R, Yin G, Li L. Assessing China’s Ecological Restoration Programs: What’s Been Done and What Remains to Be Done? *Environ Manage*. 2010;45(3):442-453. doi:10.1007/s00267-009-9387-4. | Not healthcare delivery |
| 104 | Nayaradou M, Berchi C, Dejardin O, Launoy G. Eliciting Population Preferences for Mass Colorectal Cancer Screening Organization. *Med Decis Mak*. 2010;30(2):224-233. doi:10.1177/0272989X09342747. | No implementation strategy |
| 105 | Flynn TN. Using Conjoint Analysis and Choice Experiments to Estimate QALY Values. *Pharmacoeconomics*. 2010;28(9):711-722. doi:10.2165/11535660-000000000-00000. | Commentary/Systematic Review |
| 106 | Huijps K, Hogeveen H, Lam TJGM, Huirne RBM. Preferences of cost factors for mastitis management among Dutch dairy farmers using adaptive conjoint analysis. *Prev Vet Med*. 2009;92(4):351-359. doi:10.1016/j.prevetmed.2009.08.024. | Not healthcare delivery |
| 107 | Lagarde M, Blaauw D. A review of the application and contribution of discrete choice experiments to inform human resources policy interventions. *Hum Resour Health*. 2009;7(1):62. doi:10.1186/1478-4491-7-62. | Commentary/Systematic Review |
| 108 | Cunningham CE, Vaillancourt T, Rimas H, et al. Modeling the Bullying Prevention Program Preferences of Educators: A Discrete Choice Conjoint Experiment. *J Abnorm Child Psychol*. 2009;37(7):929-943. doi:10.1007/s10802-009-9324-2. | Not healthcare delivery |
| 109 | Ryan M, Watson V, Entwistle V. Rationalising the “irrational”: a think aloud study of discrete choice experiment responses. *Health Econ*. 2009;18(3):321-336. doi:10.1002/hec.1369. | No implementation strategy |
| 110 | Wortley S, Wong G, Kieu A, Howard K. Assessing Stated Preferences for Colorectal Cancer Screening: A Critical Systematic Review of Discrete Choice Experiments. *Patient - Patient-Centered Outcomes Res*. 2014;7(3):271-282. doi:10.1007/s40271-014-0054-3. | Commentary/Systematic review |
| 111 | IJzerman MJ, van Til JA, Snoek GJ. Comparison of Two Multi-Criteria Decision Techniques for Eliciting Treatment Preferences in People with Neurological Disorders. *Patient Patient-Centered Outcomes Res*. 2008;1(4):265-272. doi:10.2165/1312067-200801040-00008. | Not healthcare delivery |
| 112 | Zeng Z, Xie Y. A preference-opportunity-choice framework with applications to intergroup friendship. *AJS*. 2008;114(3):615-648. http://www.ncbi.nlm.nih.gov/pubmed/19569394. | No implementation strategy |
| 113 | Lancsar E, Louviere J. Conducting discrete choice experiments to inform healthcare decision making: a user’s guide. *Pharmacoeconomics*. 2008;26(8):661-677. http://www.ncbi.nlm.nih.gov/pubmed/18620460. | Commentary/Systematic Review |
| 114 | Grutters JPC, Kessels AGH, Dirksen CD, van Helvoort-Postulart D, Anteunis LJC, Joore MA. Willingness to Accept versus Willingness to Pay in a Discrete Choice Experiment. *Value Heal*. 2008;11(7):1110-1119. doi:10.1111/j.1524-4733.2008.00340.x. | No implementation strategy |
| 115 | Pylypchuk Y, Selden TM. A discrete choice decomposition analysis of racial and ethnic differences in children’s health insurance coverage. *J Health Econ*. 2008;27(4):1109-1128. doi:10.1016/j.jhealeco.2007.12.001. | Not DCE |
| 116 | Fraenkel L. Conjoint Analysis at the Individual Patient Level: Issues to Consider as We Move from a Research to a Clinical Tool. *Patient*. 2008;1(4):251-253. doi:10.2165/1312067-200801040-00005. | Commentary/Systematic Review |
| 117 | Lee JT, Bridges JF, Shockney L. Can pharmacoeconomics and outcomes research contribute to the empowerment of women affected by breast cancer? *Expert Rev Pharmacoecon Outcomes Res*. 2008;8(1):73-79. doi:10.1586/14737167.8.1.73. | Commentary/Systematic Review |
| 118 | Edelmann GF, Lingevitch JF, Gaumond CF, Fromm DM, Calvo DC. Comparison of a subrank to a full-rank time-reversal operator in a dynamic ocean. *J Acoust Soc Am*. 2007;122(5):2706. doi:10.1121/1.2783127. | Not healthcare delivery |
| 119 | Jaeger SR, Cardello A V. A construct analysis of meal convenience applied to military foods. *Appetite*. 2007;49(1):231-239. doi:10.1016/j.appet.2007.02.001. | Not healthcare delivery |
| 120 | Ossa DF, Briggs A, McIntosh E, Cowell W, Littlewood T, Sculpher M. Recombinant erythropoietin for chemotherapy-related anaemia: economic value and health-related quality-of-life assessment using direct utility elicitation and discrete choice experiment methods. *Pharmacoeconomics*. 2007;25(3):223-237. http://www.ncbi.nlm.nih.gov/pubmed/17335308. | No implementation strategy |
| 121 | Lamiraud K, Geoffard P-Y. Therapeutic non-adherence: a rational behavior revealing patient preferences? *Health Econ*. 2007;16(11):1185-1204. doi:10.1002/hec.1214. | Not DCE |
| 122 | Johnson FR, Backhouse M. Eliciting Stated Preferences for Health-Technology Adoption Criteria Using Paired Comparisons and Recommendation Judgments. *Value Heal*. 2006;9(5):303-311. doi:10.1111/j.1524-4733.2006.00119.x. | No implementation strategy |
| 123 | Lancsar E, Louviere J. Deleting “irrational” responses from discrete choice experiments: a case of investigating or imposing preferences? *Health Econ*. 2006;15(8):797-811. doi:10.1002/hec.1104. | Commentary/Systematic Review |
| 124 | McIntosh E. Using discrete choice experiments within a cost-benefit analysis framework: some considerations. *Pharmacoeconomics*. 2006;24(9):855-868. http://www.ncbi.nlm.nih.gov/pubmed/16942121. | Commentary/Systematic Review |
| 125 | Johnson FR, Backhouse M. Eliciting Stated Preferences for Health-Technology Adoption Criteria Using Paired Comparisons and Recommendation Judgments. *Value Heal*. 2006;9(5):303-311. doi:10.1111/j.1524-4733.2006.00119.x. | No implementation strategy |
| 126 | D. J. Godden. Rural Health Care in the U.K.: A Rapidly Changing Scene. *J Agric Saf Health*. 2005;11(2):205-210. doi:10.13031/2013.18187. | Commentary/Systematic Review |
| 127 | Backlund EA, Stewart WP, McDonald C, Miller C. Public Evaluation of Open Space in Illinois: Citizen Support for Natural Area Acquisition. *Environ Manage*. 2004;34(5):634-641. doi:10.1007/s00267-004-0015-z. | Not healthcare delivery |
| 128 | Rodrı́guez-Mı́guez E, Herrero C, Pinto-Prades JL. Using a point system in the management of waiting lists: the case of cataracts. *Soc Sci Med*. 2004;59(3):585-594. doi:10.1016/j.socscimed.2003.11.004. | No implementation strategy |
| 129 | Zhi-feng Y, Lin-yu X. Valuing health effects from the industrial air pollution in rural Tianjin, China. *J Environ Sciences.* 16(1):157-160. http://www.jesc.ac.cn/jesc_en/ch/reader/view_abstract.aspx?file_no=20040133. | Not healthcare delivery |
| 130 | Mallawaarachchi T, Blamey RK, Morrison MD, Johnson AKL, Bennett JW. Community values for environmental protection in a cane farming catchment in Northern Australia: A choice modelling study. *J Environ Manage*. 2001;62(3):301-316. doi:10.1006/jema.2001.0446. | Not healthcare delivery |
| 131 | Bala M V, Mauskopf J. Estimating the Bayesian loss function. A conjoint analysis approach. *Int J Technol Assess Health Care*. 2001;17(1):27-37. http://www.ncbi.nlm.nih.gov/pubmed/11329843. | No implementation strategy |
| 132 | van der Pol M, Cairns J. Estimating time preferences for health using discrete choice experiments. *Soc Sci Med*. 2001;52(9):1459-1470. doi:10.1016/S0277-9536(00)00256-2. | No implementation strategy |
| 133 | Smith RD. The Discrete-choice Willingness-to-pay Question Format in Health Economics: *Med Decis Mak*. 2000;20(2):194-204. doi:10.1177/0272989X0002000205. | Commentary/Systematic Review |
| 134 | Kirkland CE. Evaluation of captioning features to inform development of digital television captioning capabilities. *Am Ann Deaf*. 1999;144(3):250-260. http://www.ncbi.nlm.nih.gov/pubmed/10423892. | Not healthcare delivery |
| 135 | Hakim Z, Pathak DS. Modelling the EuroQol data: a comparison of discrete choice conjoint and conditional preference modelling. *Health Econ*. 1999;8(2):103-116. http://www.ncbi.nlm.nih.gov/pubmed/10342724. | No implementation strategy |
| 136 | van Asseldonk MA, Huirne RB, Dijkhuizen AA. Quantifying characteristics of information-technology applications based on expert knowledge for detection of oestrus and mastitis in dairy cows. *Prev Vet Med*. 1998;36(4):273-286. http://www.ncbi.nlm.nih.gov/pubmed/9820888. | Not healthcare delivery |
| 137 | Giamalva JN, Redfern M, Bailey WC. Dietitians Employed by Health Care Facilities Preferred a HACCP System Over Irradiation or Chemical Rinses for Reducing Risk of Foodborne Disease. *J Am Diet Assoc*. 1998;98(8):885-888. doi:10.1016/S0002-8223(98)00203-X. | Not healthcare delivery |
| 138 | Chakraborty G, Woodworth G, Gaeth GJ, Ettenson R. Screening for interactions between design factors and demographics in choice-based conjoint. *J Bus Res*. 1991;23(3):219-237. doi:10.1016/0148-2963(91)90031-R. | Not empirical |
| 139 | Doessel DP. Health outcome and higher medical qualifications: an economic conception and notes on implementation. *Soc Sci Med*. 1987;24(11):897-910. http://www.ncbi.nlm.nih.gov/pubmed/3616684. Accessed March 29, 2017 | No implementation strategy |
| 140 | Wigton RS, Hoellerich VL, Patil KD. How Physicians Use Clinical Information in Diagnosing Pulmonary Embolism. *Med Decis Mak*. 1986;6(1):2-11. doi:10.1177/0272989X8600600102. | No implementation strategy |
